# Supplementary material for: Impact of functional status and biomarkers on hospital costs and readmission rates in geriatric patients: An observational study with comprehensive geriatric assessment
Source: PLoS One. 2025 May 29;20(5):e0324465. doi: 10.1371/journal.pone.0324465 (PMC12121913; doi:10.1371/journal.pone.0324465)
Supplement: S1 Table — (PDF) [file pone.0324465.s001.pdf]

SUPPLEMENTARY MATERIAL 1. APPENDIX

**Table A1. Predictors of Hospital Costs/Health Outcomes after 1 Year from discharge**	
	**Re-admission (OR)**
	1 Year
Age groups (<84, baseline):	
85 – 89	1.048
	(0.206)
90+	0.982
	(0.203)
Female	1.078
	(0.174)
Previous hospital. (6 months earlier)	5.381***
	(1.247)
Log(Length of stay)	1.556**
	(0.241)
Charlson index	1.049
	(0.058)
Number of diagnoses	0.958
	(0.053)
Respiratory Disorders, (baseline):	
Circulatory System Disorders	2.250***
	(0.476)
Digestive System Disorders	1.588
	(0.477)
Hepatobiliary & Kidney Disorders	1.308
	(0.385)
Nervous and Mental Disorders	1.059
	(0.418)
Infectious Disorders	1.064
	(0.314)
Other Disorders	1.454
	(0.469)
Barthel index category, (<=50, baseline):	
51 - 75	1.432
	(0.307)
76+	1.473
	(0.307)
Albumin category, (baseline: > 30g/l):	
<= 30 g/l	1.252
	(0.212)
Systolic Blood pressure, (baseline: >115 mmHg):	
<=115 mmHg	1.489*
	(0.259)
Observations	766
Pseudo-R2	0.099

Notes: Logit estimates for readmission within 1-year, odd ratios are reported. The Barthel index and the systolic blood pressure are considered at admission, while for readmission outcomes at discharge. *** p<.01, ** p<.05, * p<.1
